# Host Plant Specialization in the Sugarcane Aphid *Melanaphis sacchari*

**DOI:** 10.1371/journal.pone.0143704

**Published:** 2015-11-24

**Authors:** Samuel Nibouche, Stelly Mississipi, Benjamin Fartek, Hélène Delatte, Bernard Reynaud, Laurent Costet

**Affiliations:** 1 Cirad, UMR PVBMT, F-97410 Saint Pierre, La Réunion, France; 2 Université de la Réunion, UMR PVBMT, F-97410 Saint Pierre, La Réunion, France; CSIRO, AUSTRALIA

## Abstract

Most aphids are highly specialized on one or two related plant species and generalist species often include sympatric populations adapted to different host plants. Our aim was to test the hypothesis of the existence of host specialized lineages of the aphid *Melanaphis sacchari* in Reunion Island. To this end, we investigated the genetic diversity of the aphid and its association with host plants by analyzing the effect of wild sorghum *Sorghum bicolor* subsp. *verticilliflorum* or sugarcane as host plants on the genetic structuring of populations and by performing laboratory host transfer experiments to detect trade-offs in host use. Genotyping of 31 samples with 10 microsatellite loci enabled identification of 13 multilocus genotypes (MLG). Three of these, Ms11, Ms16 and Ms15, were the most frequent ones. The genetic structure of the populations was linked to the host plants. Ms11 and Ms16 were significantly more frequently observed on sugarcane, while Ms15 was almost exclusively collected in colonies on wild sorghum. Laboratory transfer experiments demonstrated the existence of fitness trade-offs. An Ms11 isofemale lineage performed better on sugarcane than on sorghum, whereas an Ms15 lineage developed very poorly on sugarcane, and two Ms16 lineages showed no significant difference in performances between both hosts. Both field and laboratory results support the existence of host plant specialization in *M*. *sacchari* in Reunion Island, despite low genetic differentiation. This study illustrates the ability of asexual aphid lineages to rapidly undergo adaptive changes including shifting from one host plant to another.

## Introduction

Most aphid genera or families are host specialized and generalist species represent only 1% of total aphid species. Some specialized aphid species are specific to a single plant species, while other genera or families are associated with a single plant genus or family [1]. Moreover, even in aphid species considered as polyphagous, sympatric populations adapted to different host plants may coexist. The existence of such host races [2] has been demonstrated in *Aphis gossypii* Glover [3]; *Acyrtocyphon pisum* Harris [4], *Myzus persicae* (Sulzer) [5] or *Schizaphis graminum* (Rondani) [6], for example. Trade-offs in fitness across hosts are regarded as one of the factors that favor such ecological specialization, according to the ‘Jack of all trades-master of none’ theory, which states that genotypes displaying highly performance in a given environment will perform poorly in other environments [7,8].

The aphid *Melanaphis sacchari* (Zehnter), is distributed worldwide, with apomictic parthenogenetic reproduction, and is mainly restricted to Poaceae hosts [9]. It is a major pest of sorghum [10] and a major virus vector in sugarcane [11]. Both sugarcane (*Saccharum* spp.) and sorghum (*Sorghum* spp.) are members of the Poaceae family and the Andropogoneae tribe. In the USA, a recent outbreak of *M*. *sacchari* has been causing serious economic damage to sorghum in the southern states since 2013 [12]. This recent change in its pest status occurred although *M*. *sacchari* has been reported in Florida since 1922 and has been a common pest of sugarcane since the late 1970s, but has never caused such outbreaks on sorghum [12–14]. This change in its pest status on sorghum raised the hypothesis [15] that these outbreaks could be due to the emergence of a new variant of sugarcane aphid that has a high preference for sorghum or to the introduction of the species *Melanaphis sorghi* (Theobald). *Melanaphis sorghi*, which has been regarded as a synonym of *M*. *sacchari* by Remaudière and Remaudière [16], is considered as a separate species by Blackman and Eastop [9], and could prefer sorghum to sugarcane [17].

The question of host plant specialization in *M*. *sacchari* has never been deeply investigated. In Brazil, Lopes da Silva et al. [18] showed that a clonal lineage derived from a female collected on sugarcane exhibited higher demographic parameters on sorghum than on sugarcane. In a previous study [19], we analyzed the worldwide genetic diversity of *M*. *sacchari*. Five multilocus lineages (MLL), or parthenogenetic lineages, were identified, each lineage grouping several multilocus genotypes (MLG) differing from each other by stepwise mutations. The distribution of the five MLLs revealed a strong geographic structuring: MLL-A in West and East Africa, MLL-B in Australia, MLL-D in the USA, MLL-E in China, and MLL-C in a wide region covering South America, the Caribbean, East Africa and the Indian Ocean. That study was carried out on samples collected on sugarcane and on three wild or cultivated *Sorghum* species. When considering the MLL–host plant association, we did not detect any effect of the host plant on genetic diversity. However, our study was carried out at a large spatial scale and considering MLL as homogeneous genetic entities, we were therefore unable to check the absence of host plant specialization at a finer spatial and genetic scale.

In the present study, to test the hypothesis of the existence of host specialized lineages in *M*. *sacchari*, we characterized its genetic diversity and its association with host plants in Reunion Island by (i) analyzing the effect of the host plant, wild sorghum or sugarcane, on the genetic structuring of populations and (ii) by performing laboratory host transfer experiment to detect trade-offs in host use.

## Material and Methods

### Insect samples

Here, an ‘individual’ refers to one individual aphid and a ‘sample’ refers a several individuals collected from the same host plant species at a given location on the same date. The complete set of individuals (S1 Table) comprised 31 samples collected from two host plants (Table 1): sugarcane (i.e. commercial cultivars derived from inter-specific crosses between *S*. *officinarum* and *S*. *spontaneum*) and wild sorghum *Sorghum bicolor* (L.) Moench subsp. *verticilliflorum* (Steud.) de Wet ex Wiersema & J. Dahlb. On Reunion Island, sugarcane is the dominant crop, cultivated on more than 50% of agricultural lands, while wild sorghum is a common ruderal weed. Sampling was carried out from 2007 to 2010. Collects were carried out during the austral summer (hot and wet season), from October to June. The geographic coordinates of sampling localities are listed in S1 Table. Most of sampling localities, except five of them (Run8, Run17, Run18, Run28, Run30), were situated within the sugarcane growing area. No specific permission was required to sample aphids in these locations. Sampling did not involve endangered or protected species. Eight of these samples (Run1 to Run8) were already included in our previous study [19].

**Table 1:** Characteristics of samples.

Sample	Locality	Date of sampling	Host plant
Run1	Sainte Marie	June, 2007	sugarcane
Run2	Etang Salé	April, 2008	sugarcane
Run3	Saint-Pierre	October, 2007	sugarcane
Run4	Savanna	April, 2008	sugarcane
Run5	Saint André	April, 2008	sugarcane
Run6	Entre Deux	March, 2009	wild sorghum
Run7	Bras Panon	January, 2010	wild sorghum
Run8	Salazie	March, 2010	wild sorghum
Run9	Sainte Marie	October, 2007	sugarcane
Run10	Sainte Marie	October, 2007	sugarcane
Run11	Saint Leu	April, 2008	sugarcane
Run12	Petite Île	April, 2008	sugarcane
Run13	Saint Philippe	April, 2008	sugarcane
Run14	Piton Sainte Rose	April, 2008	sugarcane
Run15	Entre Deux	June, 2008	wild sorghum
Run16	La Saline les Hauts	January, 2010	sugarcane
Run17	Salazie	March, 2010	sugarcane
Run18	Grande Chaloupe	January, 2010	wild sorghum
Run19	Saint Pierre	February, 2010	sugarcane
Run20	Savanna	January, 2010	wild sorghum
Run21	Saint André	January, 2010	wild sorghum
Run22	Piton Sainte Rose	February, 2010	wild sorghum
Run23	Saint Gilles les Hauts	February, 2010	wild sorghum
Run24	Sainte Anne	February, 2010	wild sorghum
Run25	Saint Pierre	February, 2010	wild sorghum
Run26	Petite Île	March, 2010	wild sorghum
Run27	Etang Salé	March, 2010	wild sorghum
Run28	Cilaos	April, 2010	wild sorghum
Run29	Saint Philippe	April, 2010	wild sorghum
Run30	Cilaos	April, 2010	wild sorghum
Run31	Sainte Anne	April, 2009	sugarcane

Only a few aphids were collected on each plant sampled to avoid collecting several individuals from the same colony. The number of plants to be sampled was not fixed and varied according to the abundance of aphid colonies. Aphids were immediately placed in 95% ethanol in Eppendorf tubes, and kept frozen at -80°C until processed.

### DNA extraction, genotyping and sequencing

#### DNA extraction and genotyping

DNA was extracted using the protocol of Sunnucks and Hales [20].

Ten microsatellite loci (Table 2) were selected among the 14 previously developed by our team for *M*. *sacchari* [21]. PCR reactions were performed with labelled primers and multiplexed as described by Nibouche et al. [19]. Genotyping was carried out using an ABI PRISM 3110 and alleles were identified at each locus by comparison with the size standard using GeneMapper version 2.5 software (Applied Biosystems).

**Table 2:** Observed microsatellite multilocus genotypes (MLG): allele size (bp) at each locus and distribution according to the host plant.

MLG	CIR-Ms-G08	CIR-Ms-G403	CIR-Ms-B03	CIR-Ms-C08*	CIR-Ms-G01	CIR-Ms-E01*	CIR-Ms-G12	CIR-Ms-G02*	CIR-Ms-E03*	CIR-Ms-D02*	Number of individuals	
											*Saccharum* sp.	*Sorghum* sp.
Ms11	233 / 233	251 / 259	213 / 213	197 / 199	185 / 210	247 / 247	212 / 216	199 / 199	186 / 193	228 / 232	120	20
Ms12	233 / 233	251 / 259	213 / 213	195 / 197	185 / 210	247 / 247	212 / 216	199 / 205	186 / 193	228 / 232	24	5
Ms13	233 / 233	251 / 259	213 / 213	197 / 199	185 / 210	247 / 247	212 / 216	199 / 201	186 / 201	228 / 232		18
Ms14	233 / 233	251 / 259	213 / 213	195 / 197	185 / 210	247 / 249	212 / 216	199 / 203	186 / 193	228 / 232	5	1
Ms15	233 / 233	251 / 259	213 / 213	197 / 199	185 / 210	247 / 247	212 / 216	199 / 201	186 / 193	228 / 232	2	253
Ms16	233 / 233	251 / 259	213 / 213	195 / 197	185 / 210	247 / 247	212 / 216	199 / 203	186 / 193	228 / 232	233	99
Ms17	233 / 233	251 / 259	213 / 213	197 / 199	185 / 210	247 / 247	212 / 216	199 / 201	186 / 193	224 / 228		2
Ms18	233 / 233	251 / 259	213 / 213	197 / 201	185 / 210	247 / 247	212 / 216	199 / 201	186 / 193	228 / 232		1
Ms19	233 / 233	251 / 259	213 / 213	195 / 197	185 / 210	247 / 247	212 / 216	199 / 203	186 / 193	220 / 228	1	
Ms20	233 / 233	251 / 259	213 / 213	195 / 197	185 / 210	247 / 247	212 / 216	199 / 203	186 / 193	228 / 230		23
Ms21	233 / 233	251 / 259	213 / 213	195 / 197	185 / 210	247 / 247	212 / 216	199 / 203	186 / 193	228 / 234	29	
Ms22	233 / 233	251 / 259	213 / 213	197 / 199	185 / 210	247 / 247	212 / 216	199 / 199	186 / 193	220 / 232	6	1
Ms23	233 / 233	251 / 259	213 / 213	197 / 199	185 / 210	247 / 247	212 / 216	199 / 199	188 / 193	228 / 232	5	

* polymorphic loci

#### Sequencing

A portion of the mitochondrial COI gene was amplified and sequenced in a total of 26 aphids. The individuals were chosen among the samples to represent at least one individual for each microsatellite multilocus genotype (MLG, see below). Two MLGs, Ms18 and Ms19, were not represented because we collected only one individual of each and their PCR amplification failed. COI fragments were amplified using the LCO1490 and HCO2198 primers designed by Folmer et al. [22]. PCR was carried out using the protocol of Kim and Lee [23]. PCR products were purified and sequenced by a subcontractor (Cogenics, Takeley, Essex, U.K.), and a consensus sequence of 658 pb was defined. Sequence alignments were performed using Geneious software version 5.6.6 [24].

### Host transfer experiment

To confirm the existence of host specialization, the development of several *M*. *sacchari* isofemale lineages belonging to different MLG was monitored on sugarcane and *S*. *verticilliflorum* plantlets in the laboratory. Four isofemale lineages were compared. An Ms11 lineage founded from individuals collected on sugarcane, an Ms15 from *S*. *verticilliflorum*, an Ms16 from sugarcane and an Ms16 from *S*. *verticilliflorum*. These are referred to as Ms11_sugarcane_, Ms15_sorghum_, Ms16_sugarcane_ and Ms16_sorghum_ respectively.

The aphids used to inoculate the plants in this experiment were obtained from laboratory clonal isofemale lineages started from females collected on sugarcane at two locations and on *S*. *verticilliflorum* at two locations. The aphids were reared in the laboratory on the same host plant as the plant on which they were collected. We used the rearing method on detached leaves described by Abu Ahmad et al. [25], at 28°C under a L12:D12 light dark regime and 80% relative humidity. Detached sugarcane leaves from cultivar MQ 76/53 were collected in the field. Detached leaves of *S*. *verticilliflorum* were collected from potted plants grown in the greenhouse. Several isofemale lineages were produced from individuals collected in the field. After establishment in the laboratory, one individual was collected from each lineage and was genotyped with the microsatellite markers as described above. The MLG of each lineage was therefore identified and only one of each lineage required (i.e. Ms11_sugarcane_, Ms15_sorghum_, Ms16_sugarcane_ and Ms16_sorghum_) was retained for the following experiment.

The experiment was performed on potted plantlets in a no-choice trial, using the experimental design described by Fartek et al. [26, 27]. The potted sugarcane plantlets were grown from stem cuttings collected in the field. Plantlets were grown in pots filled with potting soil at 25°C with a 12 hour light dark regime, until they reached the three leaf stage. The sugarcane cultivar used was ‘R570’, which is one of the two most widely grown cultivars in Reunion Island and has intermediate susceptibility to *M*. *sacchari* [27]. The *S*. *verticilliflorum* plantlets were grown from seeds collected in natural populations. Plantlets at the three leaf stage were obtained with the cultivation method described above for sugarcane.

Each host-plant × lineage modality was isolated from the others, but aphids could move from one plant to the next one within each host-plant × lineage modality. Ten apterous nymphs, aged 3–4 days (i.e., approximately 2^nd^ or 3^rd^ instar), were placed on each plantlet with a camel hair brush, and the number of aphids on the plants was scored 10 days after infestation. Three replications were carried out, set up successively on May 20, May 31, and June 14, 2011. Each replication used 12 sugarcane plantlets and 12 sorghum plantlets per isofemale lineage. Experiments were carried out at 25°C with a 12 hour light dark regime. In these conditions, the mean duration of one *M*. *sacchari* generation (adult to adult) is between 9 to 11 days and the mean longevity of individuals is between 11 and 18 days [26]. Consequently, 10 days after infestation the plantlets were colonized both by F1 progenies and by some of the initial individuals that had survived over the course of the experiment.

### Data analysis

#### Clonal diversity analysis

Single combinations of alleles were retrieved from genotyping data and arranged as unique multilocus genotypes (MLGs). Given the clonal reproduction of *M*. *sacchari*, we assumed that the different occurrences of the same MLG in a sample were the result of local clonal reproduction. We therefore kept only one representative of each MLG in each of the 31 samples for genetic and diversity analysis.

Using GENCLONE software [28], we computed a matrix of pairwise genetic distance between MLGs as the number of allelic differences between MLGs. Examination of the distribution of these distances enabled us to define a threshold below which MLGs were considered to belong to the same multilocus lineage (MLL), i.e. genotypes that differed slightly due to mutation or scoring errors according to Arnaud‐Haond et al. [29]. The same matrix of pairwise distances was also used to construct a minimum spanning network using HAPSTAR software [30]. On the set of identical loci within each MLL, we computed p_sex_, the probability that the repeated MLGs originated from distinct sexual reproductive events. A p_sex_ value lower than 0.01 supported the hypothesis that MLGs originated from the same MLL [29]. To describe clonal diversity, we computed the clonal richness index as R_MLG_ = (G-1)/(N-1), where G is the number of MLGs detected, and N is the number of samples [31].

### Prevalence of MLGs in sugarcane or sorghum

Prevalence of a MLG was defined as the proportion of samples in which at least one individual was detected. For each of the three most prevalent MLGs, i.e. Ms11, Ms15 and Ms16, a logistic model was used to analyze differences in prevalence between sorghum and sugarcane using SAS GLIMMIX procedure [32]. Because the sampling location and the host plant might be confounding effects (i.e. only one host was sampled in each location), we carried out a spatial analysis with a mixed logistic model with a G-side spherical spatial covariance structure [33]. Laplace approximation of the likelihood was used to allow model comparisons. However, the spatial correlation models could not estimate positive spatial covariance components and did not provided an improved fit (based on the AIC criterion) compared with the simple logistic model. For this reason, the results of these spatial analyses were not further taken into account. To compare the level of specialization of the three most prevalent MLGs, their prevalence ratio was computed as the ratio of the prevalence in their most preferred host to the prevalence in the least preferred host. The estimation of the prevalence ratios with their 95% confidence interval was performed with a Poisson regression with a robust error variance using the SAS GENMOD procedure [34–35].

#### Host transfer experiment

Count data from the laboratory experiment were analyzed with a generalized mixed linear model using a negative binomial distribution. The negative binomial distribution was preferred over the Poisson distribution because a strong overdispersion was obvious with the Poisson distribution model, which yielded a scaled Pearson statistic of 13.83, far from the expected 1.0 value. The lineage, the host plant, the lineage × host plant interaction and the replication (i.e. the three successive experiments) were considered as fixed effects. The 12 plantlets were considered as repeated observations and taken into account by a random lineage × host plant × replication effect. Analyses were performed using the SAS GLIMMIX procedure [32].

## Results

### Genetic and clonal diversity

A total of 855 aphids were genotyped with the 10 microsatellite markers. After retaining a single representative of each MLG in each of the 31 samples, the final dataset comprised 64 individuals (S1 Table).

**Fig 1:**
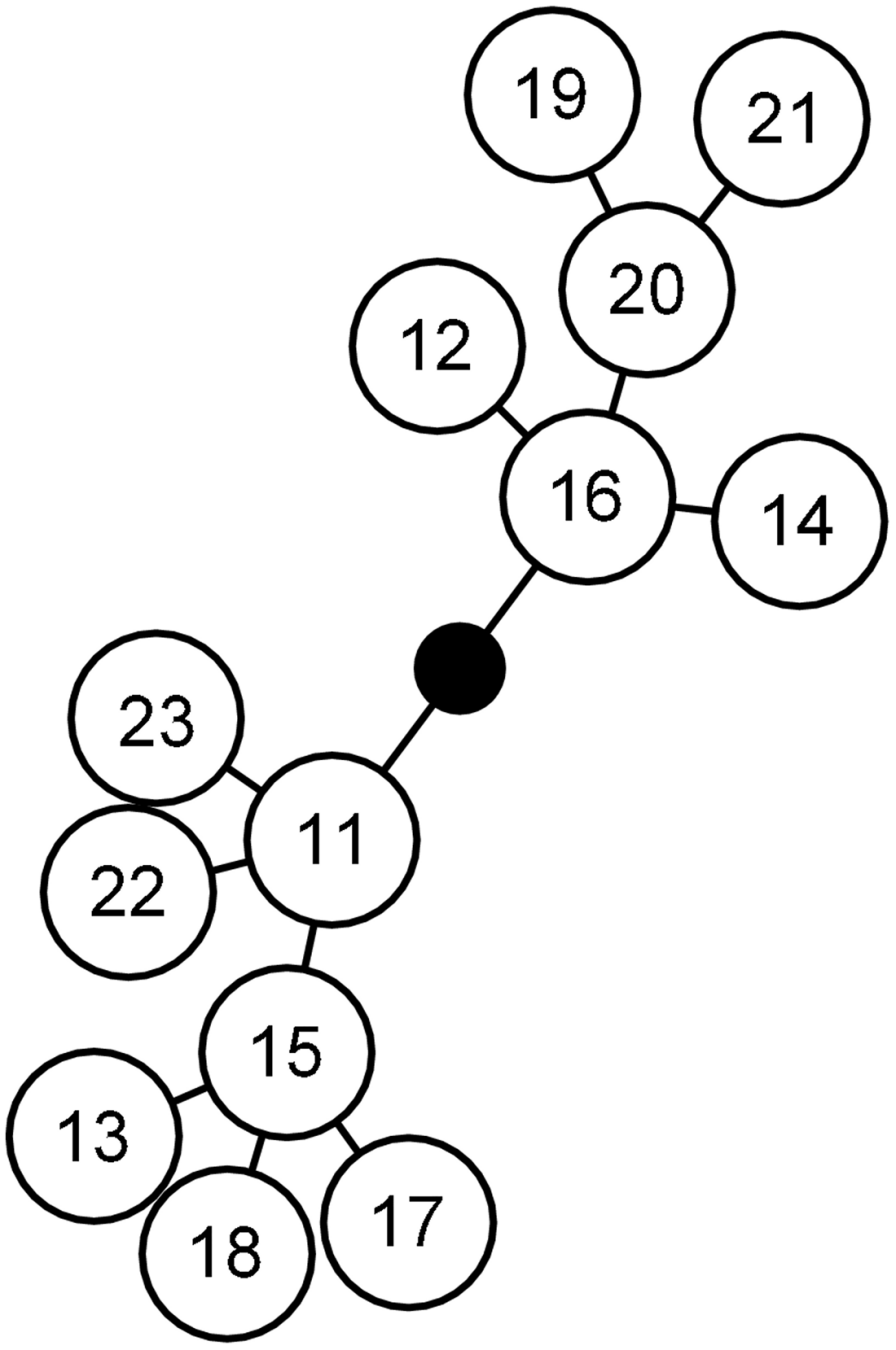
Minimum spanning network of *Melanaphis sacchari* microsatellite distances computed as the number of allele differences between MLGs. Each node represents one step in the network, i.e. a distance of one allele. The numbers in the circles refer to the numbers given to MLGs in Table 1.

Among the 10 microsatellite loci, five were polymorphic (Table 2). These five loci defined 13 MLGs. The most frequently observed MLG were Ms11 (16% of the 855 individuals), Ms15 (30%) and Ms16 (39%). The global clonal richness was low, R_MLG_ = 0.40. All pairwise numbers of distinct alleles between MLG were lower than four, and the distribution was unimodal (S1 Fig). The value of p_sex_ was <0.001, which confirmed that the 13 MLGs belonged to the same multilocus lineage (MLL), i.e. genotypes that differed slightly due to step mutations or scoring errors (Fig 1).

Among the 26 COI sequences obtained (GenBank accessions listed in S1 Table), a single haplotype was observed.

### Host plant specialization

**Fig 2:**
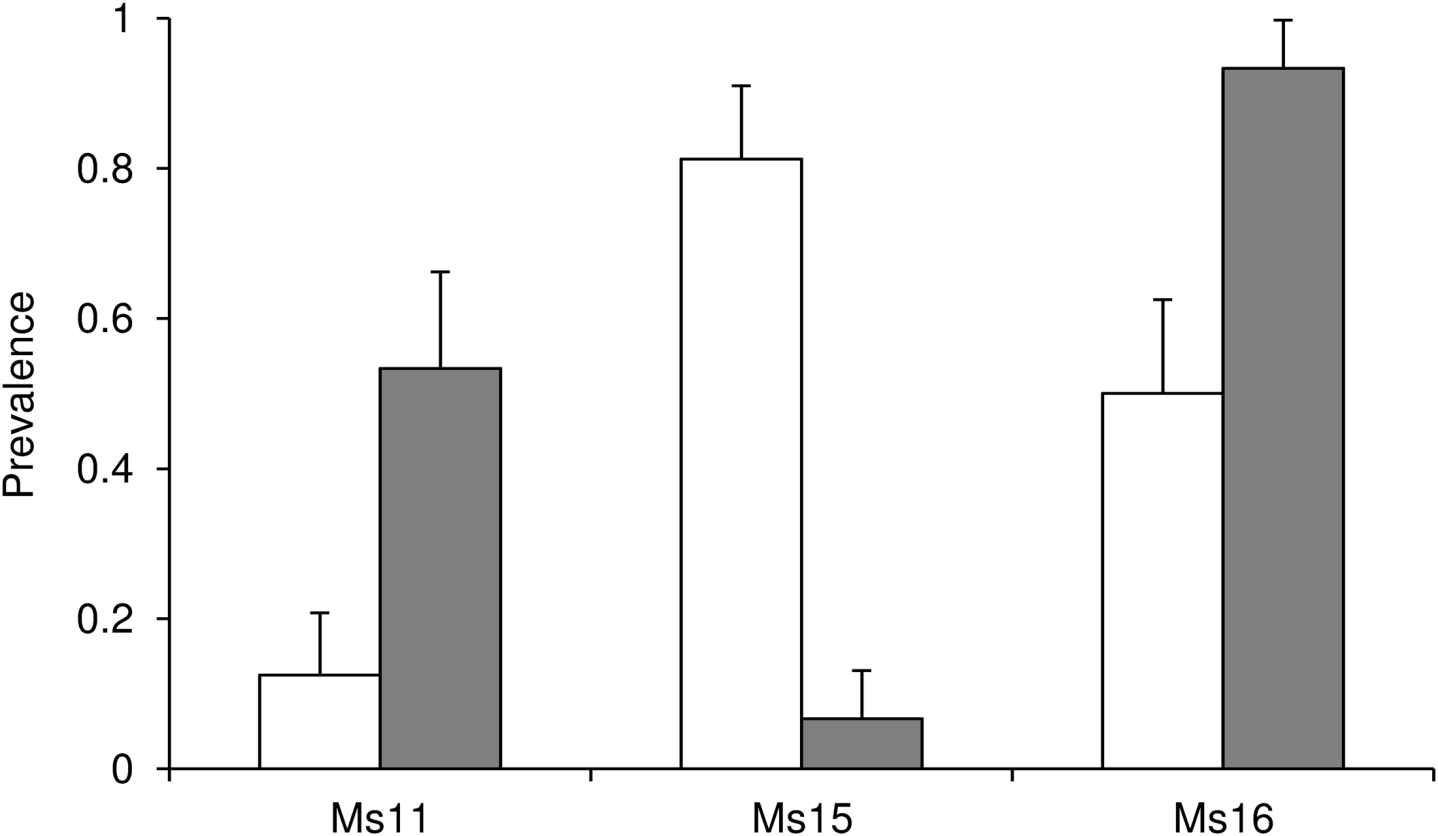
Prevalence of the three most frequent MLGs on sorghum (white bars) and sugarcane (grey bars) samples. Vertical lines represent standard errors of prevalences.

The number of sorghum or sugarcane samples hosting each MLG is summarized in Table 3. Three MLGs (Ms11, Ms15 and Ms16) were frequent enough to allow a statistical comparison of their prevalence in sorghum vs. sugarcane samples (Fig 2). All the results of the tests were significant. Ms11 (F = 5.15; df = 1, 29; P = 0.0308) and Ms16 (F = 5.27 df = 1, 29; P = 0.0291) showed higher prevalence in sugarcane samples than in sorghum samples. Conversely, Ms15 showed a significantly higher prevalence in sugarcane than in sorghum (F = 11.38; df = 1, 29; P = 0.0021). The estimated prevalence ratios [95% CI] in favor of sugarcane for Ms11 and Ms16 were respectively 4.27 [1.07; 16.96] and 1.87 [1.12; 3.10]. For Ms15, the prevalence ratio in favor of sorghum was 12.19 [1.80; 80.14]. The large confidence intervals due to the small number of samples (31), prevented us from ranking the prevalence ratios to rank the specialization of the three MLGs.

**Table 3:** Number of samples hosting at least one individual of each of the 13 MLGs on sugarcane or sorghum samples.

MLG	Number of samples hosting each MLG	
	sorghum	sugarcane
Ms11	2	8
Ms12	2	3
Ms13	1	0
Ms14	1	1
Ms15	13	1
Ms16	8	14
Ms17	1	0
Ms18	1	0
Ms19	0	1
Ms20	1	0
Ms21	0	2
Ms22	1	1
Ms23	0	2
Total number of samples	16	15

**Fig 3:**
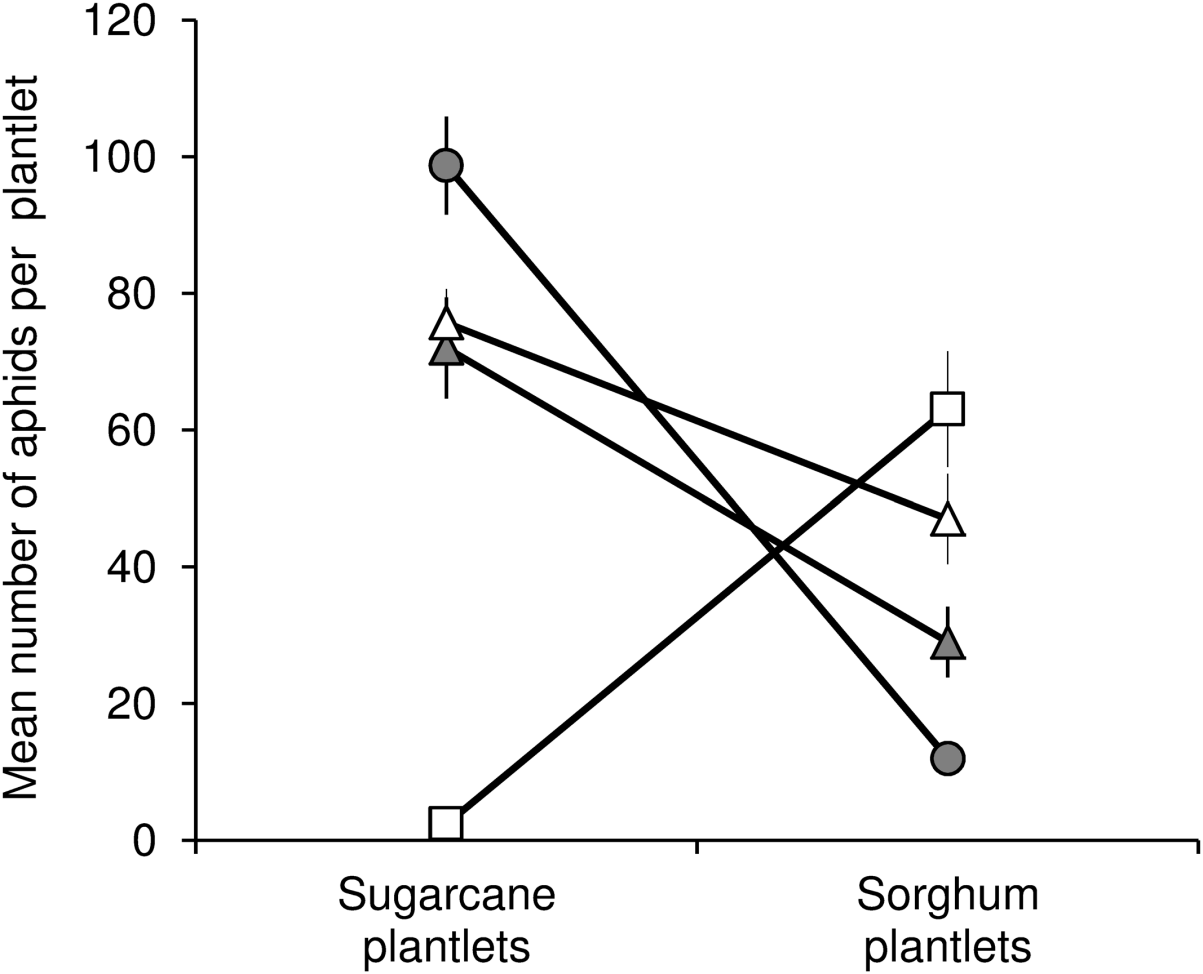
Laboratory comparison of fitness trade-offs in three MLGs. Ms11 (circles), Ms15 (squares), or Ms16 (triangles). Host transfer experiments were carried out with four isofemale lineages derived from individuals collected either on sorghum (white symbols) or on sugarcane (grey symbols). The figure represents the mean number of aphids per sugarcane or sorghum plantlet 240 hours after 12 plantlets had each been infested with 10 individuals. The experiment was repeated three times. Vertical lines represent the standard errors of means.

The results of the host transfer experiment are illustrated in Fig 3. At 240 hours post infestation, the lineage × host plant effect was significant (F = 19.31; df = 3, 14; P < 0.0001) and revealed a significant host specialization. The Ms11_sugarcane_ aphid population size was significantly lower on sorghum than on sugarcane (t = 3.69; df = 14; P = 0.0024). Conversely, the Ms15_sorghum_ population size was significantly higher on sorghum than on sugarcane (t = 6.22; df = 14; P < 0.0001). No significant difference in Ms16_sorghum_ was observed between sugarcane and sorghum (t = 1.20; df = 14; P = 0.2488). The difference in Ms16_sugarcane_ was close to the significance level but remained non-significant (t = 2.11; df = 14; P = 0.0532).

Ms15_sorghum_ appeared to be more specialized than Ms11_sugarcane_. The population of Ms15_sorghum_ on sugarcane decreased 4.2-fold, from 10 aphids per plantlet at the beginning of the experiment to 2.36 ± 0.73 aphids per plantlet 240 hours after infestation. On sorghum, the Ms11_sugarcane_ population remained stable over the course of the experiment, increasing slightly from 10 to 11.97 ± 1.94 aphids per plantlet. At 240 hours, the population size of Ms11_sugarcane_ on sorghum was significantly higher than the population of Ms15_sorghum_ on sugarcane (t = 3.48; df = 14; P = 0.0037), suggesting a higher level of specialization by Ms15 than by Ms11.

**Fig 4:**
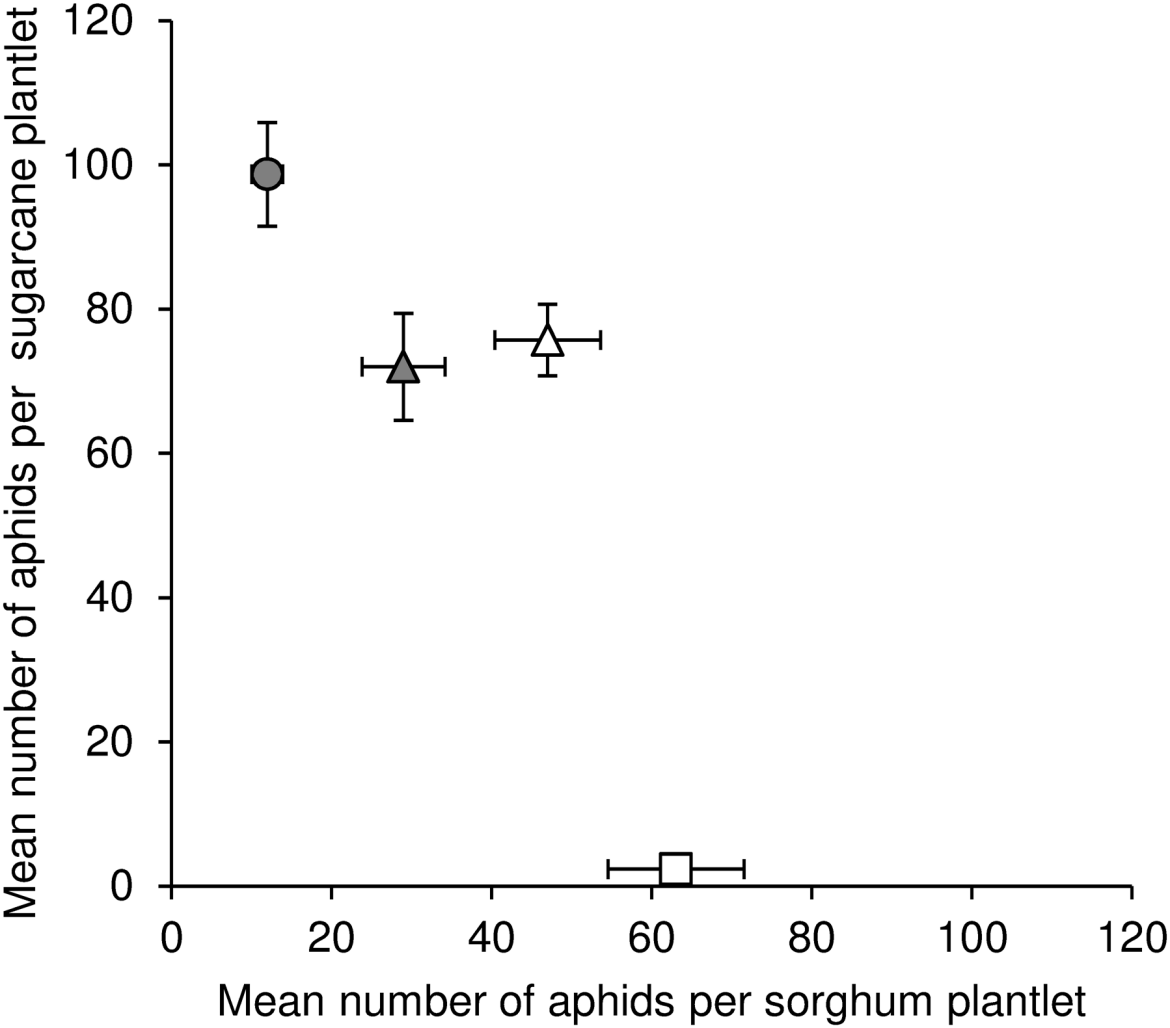
Illustration of the negative correlation of the performances on sorghum and sugarcane of four isofemale lineages in the laboratory host transfer experiments. The lineages belong to three MLGs: Ms11 (circles), Ms15 (squares), or Ms16 (triangles). Vertical and horizontal bars represent the standard errors of means.

Fig 4 presents the results of the host transfer experiment illustrating the negative correlation of fitness on sugarcane and fitness on sorghum.

## Discussion

In this study, the genetic diversity of *M*. *sacchari* in Reunion Island was represented by 13 MLGs which differed from one another by stepwise mutations. Three of the 13 MLGs, (Ms11, Ms16 and Ms15), were the most frequent. Among these 13 MLGs, only five (Ms11, Ms12, Ms15, Ms16, Ms21) had already been observed in our previous study [19]. The eight newly observed MLGs were rare and were only observed in one or two samples. This increase in the number of observed MLGs is the result of the increase in the number of samples, not to higher clonal diversity, as shown by the R_MLG_ of 0.40, which is similar to the value observed previously at the world scale (0.37). The 13 MLGs observed in La Reunion belong to a unique multilocus lineage MLL-C, previously detected in South America, the Caribbean, East Africa and the Indian Ocean [19]. The COI haplotype is also the only one we observed previously in the individuals belonging to this MLL-C [19].

Both field and laboratory results support the hypothesis of host plant specialization in *M*. *sacchari* in Reunion Island. The genetic structure of the populations was linked to the host plants. Ms11 and Ms16 were significantly more frequently observed on sugarcane, while Ms15 was almost exclusively collected in colonies on the wild sorghum *S*. *verticilliflorum*. Laboratory transfer experiments demonstrated the existence of fitness trade-offs between the two host plants, and showed that fitness on sugarcane is negatively correlated to that on sorghum. Ms11 performed better on sugarcane than on sorghum, while the opposite was the case of Ms15. Ms16 was intermediate, its field prevalence was significantly higher on sugarcane than on sorghum, and its performance was intermediate on both hosts in the laboratory.

Although some authors demonstrated that conditioning or experience (i.e. the host plant on which aphids were reared before the experiment) does not interfere with the genetic based specialization [36], other studies demonstrated that conditioning or experience can alter the interpretation of host transfer experiments [37–38]. In our study, the four lineages were reared on the plant from which they originated and we did not include a conditioning step on another Poaceous host previous to the transfer experiments. Nevertheless, our results do not suggest a pronounced experience effect in the two Ms16 lineages collected and reared either on sorghum or on sugarcane in that no significant reduction in fitness was observed when they were transferred on the alternate host.

Host specialization has been demonstrated in several aphid species. In *Aphis gossypii*, Margaritopoulos et al. [39] reported morphometric differences between aphids originating from Compositae and those collected on Cucurbitaceae and Malvaceae. A study by Carletto et al. [3] demonstrated the existence of five *A*. *gossypii* host races specialized on Cucurbitaceae, cotton, eggplant, potato and chili or sweet pepper. In the *Acyrthocyphon pisum* complex, 11 well distinguished sympatric populations were also demonstrated on several Fabaceae species [4]. In *Schizaphis graminum*, analysis of COI revealed three clades whose host partitioning suggested the existence of host races [6]. Host races were also identified on several Poaceae hosts in *Sitobion avenae* [40–42]. In these four species, the existence of host races is associated with a strong genetic structuring revealed by DNA sequence variations in barcoding regions or by Bayesian clustering analysis of microsatellite data. In contrast, our study revealed strong host specialization despite low genetic differentiation. The Ms15 lineage was seen to be specialized on sorghum although it differed only by one allele in the CIR-Ms-G02 locus from the lineage Ms11, which developed better on sugarcane than on sorghum. This low genetic differentiation suggests that the appearance of the sorghum specialized host race Ms15 could be recent. On the other hand, Ms16 appears as intermediate, with a partial adaptation to sorghum, although Fig 1 shows that Ms16 is genetically closer to Ms11 than to Ms15, which could suggest that the partial adaptation of Ms16 to sorghum is independent from the adaptation of Ms15. Further experiments could aim at exploring the relationship between genetic diversity and host adaptation by characterizing the adaptation of the less frequent MLGs.

This study provides an example of the evolution of an ecological trait in a species with strictly parthenogenetic reproduction; such examples challenge the view that parthenogenesis is an evolutionary dead end and show that asexual aphid lineages can rapidly undergo adaptive changes including host plant shifts [43].

## Supporting Information

S1 Fig Distribution of the pairwise number of different alleles between MLGs. (PDF)

S1 Table Voucher number, sampling information, GenBank accession and SSR genotyping of individual aphids. (XLSX)
