# Supplementary figures and images for: Host Plant Specialization in the Sugarcane Aphid Melanaphis sacchari
Source: PLoS One. 2015 Nov 24;10(11):e0143704. doi: 10.1371/journal.pone.0143704 (PMC4658203; doi:10.1371/journal.pone.0143704)

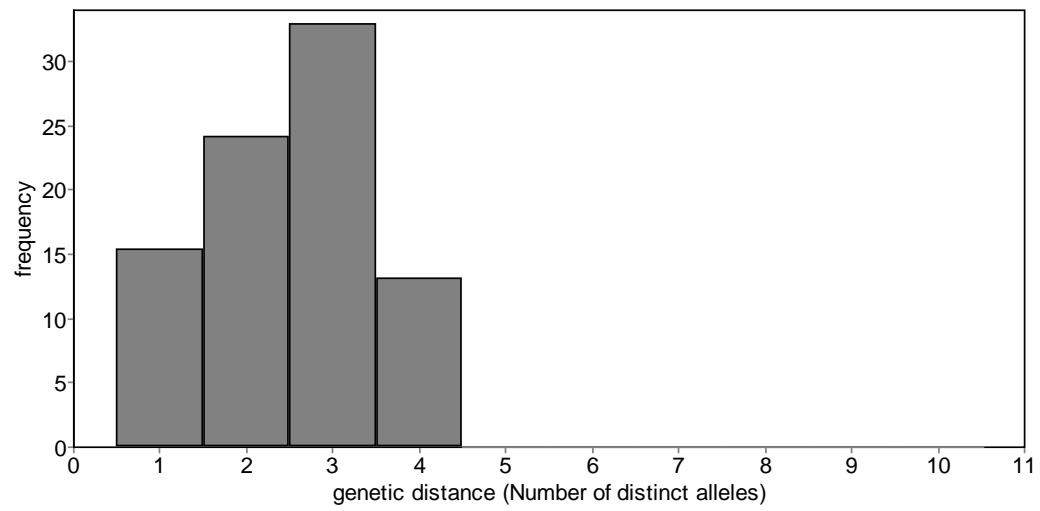

**Figure S1.** Distribution of the pairwise number of different alleles between MLGs.

Supplement: S1 Fig — (PDF) [file pone.0143704.s001.pdf]
